# Clinical observation of fallopian tube obstruction recanalization by ozone

**DOI:** 10.12669/pjms.332.11961

**Published:** 2017

**Authors:** Niuniu Sun, Lequn Wei, Diansen Chen, Wanqin Gao, Huanzhang Niu, Chao He

**Affiliations:** 1 Niuniu Sun, Department of Humanities, School of Nursing, Henan University of Science and Technology, Luoyang 471023, China; 2 Lequn Wei, Shaanxi University of Chinese Medicine, Xianyang 712046, China., The First Affiliated Hospital, and College of Clinical Medicine of Henan University of Science and Technology, Luoyang 471003, China; 3 Diansen Chen, The First Affiliated Hospital, and College of Clinical Medicine of Henan University of Science and Technology, Luoyang 471003, China; 4 Wanqin Gao, The First Affiliated Hospital, and College of Clinical Medicine of Henan University of Science and Technology, Luoyang 471003, China; 5 Huanzhang Niu, The First Affiliated Hospital, and College of Clinical Medicine of Henan University of Science and Technology, Luoyang 471003, China; 6 Chao He, Second Affiliated Hospital of Shaanxi University of Chinese Medicine, Xianyang 712000, China., Shaanxi University of Chinese Medicine, Xianyang 712046, China

**Keywords:** Tubal infertility, Fallopian tube obstruction, Ozone, Interventional therapy

## Abstract

**Objective:**

To investigate the clinical effect of fallopian tube obstruction recanalization by ozone.

**Methods:**

Total 116 cases of patients undergoing the fallopian tube obstruction were randomly divided into the experimental group and control group, with 58 cases in each group. All patients underwent the interventional fallopian tube recanalization. The patients in the experimental group underwent the fallopian tube perfusion by the ozone water. Meanwhile, the patients in the control group were treated with the conventional anti-inflammatory and anti-adhesion drugs. After the follow-up visit for 6 months, the pregnancy rate and fallopian tube re-occlusion rate were counted and compared between the two groups. Meanwhile, the symptoms were evaluated and compared between the two groups after the operation for two weeks.

**Results:**

The success rate of fallopian tube recanalization was 93.1% (54/58), the pregnancy rate was 79.3% (46/58) and the recurrence rate was 5.2% (3/58) in the experimental group. While the success rate of fallopian tube recanalization was 91.4% (53/58), the pregnancy rate was 60.3% (35/58) and the recurrence rate was 17.2% (10/58) in the control group. Analysis showed that there was no significant difference in the recanalization success rate between the two groups (P>0.05). However, the pregnancy rate and re-occlusion rate in the experimental group were significantly lower than those of the control group (P<0.05), and the difference was statistically significant. There was no significant difference in the discomfort symptoms between the experimental group and control group (P>0.05).

**Conclusion:**

Fallopian tube recanalization by ozone perfusion can effectively increase the postoperative pregnancy rate and reduce the fallopian tube re-occlusion.

## INTRODUCTION

Fallopian tube factor is one of the most common causes of infertility.^1^ For the multipara, the fallopian tube adhesion and obstruction induced by the tubal, uterine and pelvic inflammation are relatively common. Inflammatory stimulation can cause the fallopian tube wall stiffness, peripheral tissue adhesion and lumen obstruction, so that it is difficult to pick up the ovum and delivery the sperm, and thus inducing the secondary infertility.^2^

At present, in order to adjust the population policy in China, the full liberalization of the second child policy was formally implemented in January 1st 2016, which means that a couple can give birth to two babies. The multipara has strong desires of childbearing again. How to effectively recanalize the obstructed fallopian tube becomes an important clinical problem for the pregnancy again. The fallopian tube interventional recanalization is an effective operation for treating the fallopian tube obstruction.^3^-^6^ However, the obstruction easily recurs. At present, the ozone has been applied in the treatment of gynecological infectious diseases at home and abroad for decades. It has achieved good curative effect for all kinds of vaginitis, cervicitis, salpingitis, endometritis and pelvic inflammatory disease.^7^ However, it has no toxicity for the normal tissue. It can change the pH value of female genital duct, but will not cause dysbacteriosis or drug resistance.^8^

In recent years, the ozone water perfusion has been performed in the fallopian tube interventional rehabilitation in our hospital,^9^ which has effectively reduced the patients’ re-occlusion. Our objective was to investigate the clinical effect of fallopian tube obstruction recanalization by ozone.

## METHODS

Total 116 patients with fallopian tube obstruction admitted in our hospital were selected from March 2015 to November 2015. They were confirmed by clinical practice and salpingography. The patients were randomly divided into the experimental group and control group, with 58 cases in each group. The patients in the experimental group aged 24-37 years old and the average age was (28.4±3.1) years; including 17 cases of unilateral obstruction and 41 cases of bilateral obstruction; 51 cases of left obstruction and 48 cases of right obstruction; Obstruction site: 24 cases of is thmusobstruction and 34 cases of interstitial obstruction; Previous reproductive history: 56 cases of first childbearing, 2 cases of second childbearing. The patients in the control group aged 25-38 years old and the average was (28.7±3.6) years; including 18 cases of unilateral obstruction and 40 cases of bilateral obstruction; 50 cases on the left side, 48 cases on the right side; Obstruction site: 22 cases was of is thmusobstruction and 36 cases of interstitial obstruction; Previous reproductive history: 57 cases of first childbearing and one case of second childbearing. There was no significant difference between the two groups (P>0.05) (Table-I). This study was conducted in accordance with the declaration of Helsinki. This study was conducted with approval from the Ethics Committee of the Second Affiliated Hospital of Shaanxi University of Chinese Medicine. Written informed consent was obtained from all participants.

**Table-I:** Comparison of the clinical data between the two groups.

*Groups*	*Age*	*Obstruction site*				*Reproductive history*	

		*Unilateral*	*Bilateral*	*Thmus*	*Interstitial*	*First childbearing*	*Second childbearing*
Experimental group	28.4±3.1	17	41	24	34	56	2
Control group	28.7±3.6	18	40	22	36	57	1
p	0.6315	0.8397		0.7042		0.5586	

### Inclusion criteria

(1) Multipara; (2) the patients and their family member singed the informed consent; (3) the fallopian tube obstruction was confirmed by clinical practice and salpingography.

### Exclusion criteria

(1)Acute internal and external edeitis, but was not controlled; (2) menstrual period; (3) the patients could not tolerate the operation because of severe systemic diseases or physical conditions; (4)fallopian tube re-occlusion; (5)abortion or curettage in six weeks.

### Methods

**Fig.1:**
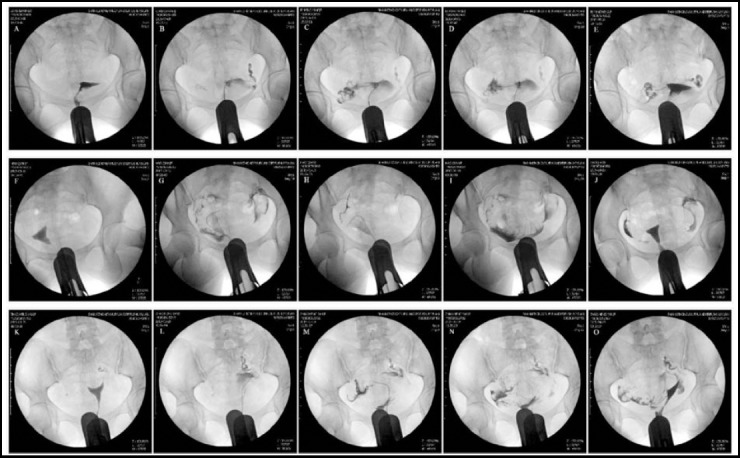
The process of the fallopian tube recanalization in multipara by ozone water perfusion. **A, F, K:** cervix, uterine cavity and salpingography showed bilateral fallopian tube obstruction; **B, G, L:** the guide wire was used to dredge the left fallopian tube.1-2ml contrast agent was injected to determine the unobstructed fallopian tube; **C, H, M:** the right fallopian tube was dredged using the guide wire. 1-2ml contrast agent was injected to determine the unobstructed fallopian tube. **D, I, N:** the uterine cavity and bilateral fallopian tubes were perfused using 20ml 40ug/ml ozone water. **E, J, O:** after the fallopian tube obstruction recanalization then the first menstrual cycle for 3-7 d, the uterus and bilateral fallopian tubes radiography was performed under the digital subtraction X ray machine. The result showed that obstruction of bilateral fallopian tubes were removed.

All patients underwent the interventional fallopian tube recanalization. The operation process was as shown in Fig.1: after menstruation ended for 3-7 days, the patient was in the lithotomy position, the cervix was fully exposed using the bivalve speculum. The anterior lip cervix was clamped. The 10F guide tube with gasbag (Cook, Bloomington, Ind, USA) was inserted into the anterior end into the uterine cavity through the cervix. The guide tube was fixed and the air bag was expanded. The 5F guide tube was selectively inserted into the interstitial fallopian tube and the salpingography was performed, so as to determine the occlusion site. The micro guide wire and micro guide tube were replaced, gently moved back and forth in the obstructed fallopian tube, so as to dredge the lumen. Meanwhile the liquid was used to fill the lumen. The operation should be step by step and gradually push inside until the obstruction in fallopian tube was removed. The second salpingography confirmed the lumen was unobstructed. The affected fallopian tube was treated with the conventional hydrotubation, so as to eliminate the inflammation and avoid the re-adhesion. The patients in the control group were perfused with dexamethasone 5mgα-chymotrypsin 4000 U+gentamicin 80,000 U+ contrast agent 5 ml+ appropriate normal saline, a total of 20 ml. In the experimental group, 20ml 40ug/ml ozone water was prepared using the ozone medical apparatus (Fumener, Jiangmen, China) to perfuse the uterine cavity and bilateral fallopian tubes. The salpingography was performed using the digital subtraction angiography machine (GE Healthcare, Bethesda, MD, USA) after the first menstrual cycle end for 3-7 d. The fallopian tube was examined. The sexual intercourse could begin after the postoperative 2-3 menstrual cycles.^10^

### Observation index

The discomfort symptoms were evaluated and compared in the postoperative two weeks between two groups. The patients were followed up for six months. The pregnancy rate and fallopian tube re-occlusion rate were observed and analyzed in the two groups.

### Discomfort symptom assessment

The discomfort symptoms included fever, abdominal pain and vaginal bleeding. If the above symptoms were mild and lasted for less than four days, the drugs or other special treatments were not required, the symptoms were assessed as Grade-1; If the above symptoms were lasted for 4-7 days, the symptoms were assessed as 2; If the above symptoms were lasted for over7 d, the symptoms were assessed as Grade-3. If the above symptoms were obvious and the drug therapy was required, the duration was less than 4 four days, the symptoms were assessed asGrade-3. If the duration was 4~7 days, the symptoms were assessed as Grade-4. If the duration was more than seven days, the symptoms were assessed as Grade-5. If the patients appeared to fever, severe abdominal pain, regardless of duration, the symptoms were assessed as Grade-5.^4^

### Statistical analysis

All data were analyzed using the SPSS19.0 statistical software (SPSS Inc, Chicago, IL, USA). The measurement data were compared using the t test. The count data were compared using the X^2^ test. P<0.05 indicated that the difference was statistically significant.

## RESULTS

### The therapeutic effect of operation

The success rate of fallopian tube recanalization was 93.1% (54/58) in the experimental group, while the control group was 91.4% (53/58). There was no significant difference between the two groups (P>0.05) (Table-II).

**Table-II:** Comparison of clinical efficacy between the two groups % (n).

*Groups*	*The success rate of recanalization*	*The pregnancy rate*	*The re-occlusion rate*
Experimental group	93.1(54/58)	79.3(46/58)	5.2(3/58)
Control group	91.4(53/58)	60.3(35/58)	17.2(10/58)
P	0.7285	0.0261	0.0394

### Follow-up results

After the follow-up visit for 6 months, the pregnancy rate was 79.3% (46/58) in the experimental group. The salpingography of the unpregnant women showed that three cases had re-occlusion and the re-occlusion rate was 5.2% (3/58). While the pregnancy rate was 60.3% (35/58) in the control group and the re-occlusion rate was 17.2% (10/58). The analysis showed that the pregnancy rate and re-occlusion rate in the experimental group were significantly lower than those of the control group (P<0.05), and the difference was statistically significant (Table-II).

### Discomfort symptoms

In the experimental group, 17 cases occurred to low-grade fever and six cases occurred to abdominal pain. Among them, the abdominal pain was obvious in one case of patient; The average score of discomfort symptoms was (1.7±0.8). In the control group, 20 cases occurred to low-grad two cases; The average score of discomfort symptoms was (2.0±0.9). There was no significant difference between the two groups (P>0.05).

## DISCUSSION

Fallopian tube obstruction, also known as tubal infertility, is one of the main causes of infertility in women of childbearing age.^11^ There are many reasons for fallopian tube occlusion. The common causes include pelvic, vaginal and peripheral organ inflammation. If the above inflammations are not controlled in time, the upward diffusion will appear, involving the fallopian tubes, resulting in fallopian tube mucosal swelling, vasodilatation and congestion, fibrin exudation, adhesion with surrounding tissues,^12^-^14^ leading to tubal obstruction and infertility. In addition, feculent sexual intercourse, sexual intercourse during menstruation, long-term vaginal bleeding, abdominal surgery and long-term sitting can cause the fallopian tube occlusion.

Fallopian tube interventional recanalization is effective for the recanalization of the lumen, which has good curative effect for isthmus or interstitial occlusion.^15^ This surgery has many advantages, including less injury, safe operation and quick recovery. It has replaced the traditional laparotomy and has been widely used in clinical practice. The shortcoming is the radiation hazards of X-ray. The patients accept a small amount of radiation. Considering better prenatal and postnatal care, the sexual intercourse should be performed after the postoperative 2-3 menstrual cycles. However a considerable number of patients still appeared to the postoperative re-occlusion, which affects the efficacy of surgery. The reason for re-occlusion may be as follows: (1) after adhesiolysis, the wound surface occurs to inflammatory exudation; (2) the fallopian tube is scratched by technical reasons; (3) the intraoperative conventional drug perfusion only has the transient anti-inflammatory and anti-adhesion effects.^16^ The efficacy is not sustained.

Ozone is non-toxic and innocuous. It can efficiently sterilize and treat various diseases, which is widely applied in the whole world.^17^-^20^ Moreover, the ozone has strong oxidizing ability, can inhibit prostaglandin synthesis, antagonize the release of inflammatory factors, reduce the local edema exudation and tissue hypoxia, improve the local cycle through inducing the overexpression of antioxidant enzymes. The above effects can promote the wound repair of the fallopian tube, and then reduce the obstruction again.^21^ The results showed that compared with the conventional gentamicin, glucocorticoid drug and other perfusion drugs, although the recanalization success rates showed no significant difference between the two groups, the postoperative pregnancy rate after ozone perfusion showed significant advantages. Postoperative follow-up data showed that the recurrence rate of the ozone therapy was lower, showing that the ozone perfusion was helpful to decrease the postoperative adhesion and improve the pregnancy rate.

The evaluation of postoperative discomfort symptoms showed some patients appeared to have fever, abdominal pain and other symptoms in the two groups. However, the fallopian tube perforation and other severe injuries did not appear. The above symptoms disappeared after being positively treated.

In short, fallopian tube obstruction is one of the main causes of infertility. The ozone perfusion was performed in the multipara with fallopian tube obstruction in this study. The results showed that ozone was helpful to improve the postoperative pregnancy rate and reduce the re-occlusion. Ozone not only has the characteristics of no drug resistance and low cost, but also has no obvious adverse reaction in the perioperative period and better therapeutic safety. Combined with the above advantages, it is worth popularizing in the clinical treatment of fallopian tube obstruction in the multipara.

### Authors’ Contribution

**HC** conceived, designed and did statistical analysis & editing of manuscript.

**WLQ, SNN, CDS, CWQ and NHZ** did data collection and manuscript writing.

**HC** takes the responsibility and is accountable for all aspects of the work in ensuring that questions related to the accuracy or integrity of any part of the work are appropriately investigated and resolved.
